# Big Losses Lead to Irrational Decision-Making in Gambling Situations: Relationship between Deliberation and Impulsivity

**DOI:** 10.1371/journal.pone.0009368

**Published:** 2010-02-23

**Authors:** Yuji Takano, Nobuaki Takahashi, Daisuke Tanaka, Naoyuki Hironaka

**Affiliations:** 1 SHIMOJO Implicit Brain Function Project, Exploratory Research for Advanced Technology, Japan Science and Technology Agency, Atsugi-shi, Kanagawa, Japan; 2 Department of Regional Education, Faculty of Regional Sciences, Tottori University, Tottori-shi, Tottori, Japan; University of Wuerzburg, Germany

## Abstract

In gambling situations, we found a paradoxical reinforcing effect of high-risk decision-making after repeated big monetary losses. The computerized version of the Iowa Gambling Task (Bechara et al., 2000), which contained six big loss cards in deck B', was conducted on normal healthy college students. The results indicated that the total number of selections from deck A' and deck B' decreased across trials. However, there was no decrease in selections from deck B'. Detailed analysis of the card selections revealed that some people persisted in selecting from the “risky” deck B' as the number of big losses increased. This tendency was prominent in self-rated deliberative people. However, they were implicitly impulsive, as revealed by the matching familiar figure test. These results suggest that the gap between explicit deliberation and implicit impulsivity drew them into pathological gambling.

## Introduction

Human decision-making is not always rational. We sometimes behave irrationally even if we ponder what to do and not to do. Certainly, our decision-making is influenced by our own cognitive styles and personality traits. Impulsivity is an important factor that biases our evaluation of cost and benefit and subsequent decision-making. A lot of experimental studies show that impulsive persons prefer immediate small rewards or even adverse outcomes to delayed large rewards [1]–[4]. Impulsivity is thought to be closely related to addictive behaviors, such as illicit drug use and pathological gambling [5]–[8]. However, it is unknown whether impulsivity is a constant behavioral trait or not. Are there “impulsive” persons and “deliberate” persons? Do “impulsive” persons always behave impulsively? It is likely that there is dissociation between conscious reasonable thinking and the unconscious “implicit” origin of actual decision making.

For example, using the Iowa gambling task (IGT), which is an experimental tool to investigate risk-taking decision making, somatic markers, such as palpitation or diaphoresis, precede a person's behavioral switch from risky to cautious choices [9]–[10]. The IGT is a card selection task in which participants are required to choose one card at a time from four card decks. Each card depicts imaginary monetary gain or loss. Participants are encouraged to increase their monetary gain. Two of the four card decks are high-risk/high-return (deck A & B), while the other two decks are low-risk/low-return (deck C& D). It is well known that the normal healthy persons shift their card selection from high-risk/high-return decks to low-risk/low-return decks. Persisting in high-risk/high-return card choices is known to represent impulsivity and to relate to brain injuries [9]–[12], psychiatric diseases [13]–[16], and substance abuse [17]–[20].

Previous studies analyzing the high-risk decks revealed a phenomenon related to deck B (called ‘prominent deck B’)[21]. Some normal subjects preferred deck B to the good final-outcome decks C or D. This preference had not been apparent in studies that used the sum of decks A and B [21]. Consequently, in the present study, we focused on deck B's effect on irrational decision-making and examined the relationship between selection of card decks and impulsivity. Our overall goal is to reveal the triggers of irrational decision-making in normal people and how personality traits relate to it. For this purpose, we used a computer version of IGT that makes progressive changes in delayed punishment (the decks of this new version are denoted as A', B', C', and D') [10]. This version has more drastic monetary changes than in the original version (the original decks are denoted without dashes).

In experiment 1, we examined the relationship between behavior in the gambling task and cognitive reflection or impulsivity [22], sensation seeking [23]–[24] and trait anxiety [25]–[26]. In experiment 2, we examined the relationship between behavior in the gambling task and multiple personality traits (Neo-PI-R) [27]–[28] and conducted a behavioral test of impulsivity, the Matching Familiar Figure Test (MFFT) [29].

## Results

### Experiment 1

#### Card selection

**Figure 1:**
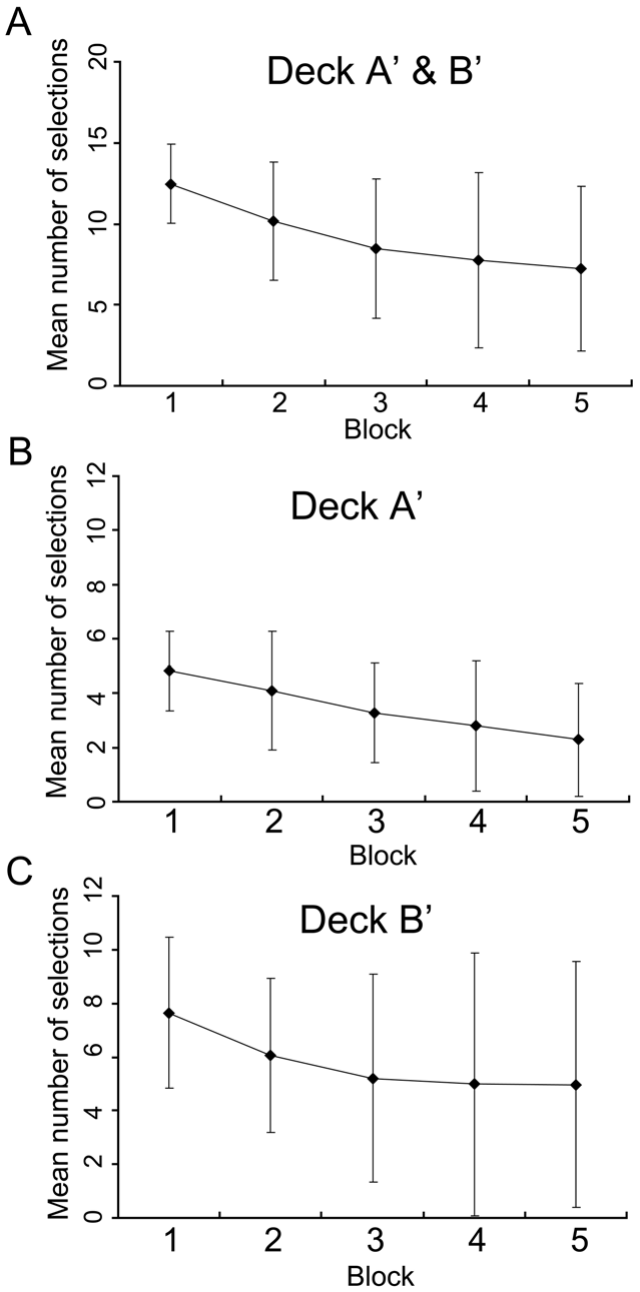
Mean number of high-risk/high-return choices across trials along with standard deviations. A total of 100 trials were divided into 5 blocks of 20 trials. The total number of selections from deck A' and deck B' decreased across trials (A). Selections from deck A' monotonously decreased (B). However, selections from deck B' initially decreased but later did not decrease. (C). Moreover, towards the end of the IGT, the deviations became larger regarding selections from deck B'.

Consistent with previous findings, high-risk/high-return choices (selections from deck A' and deck B') decreased across trials. We defined 20 card selections to be 1 block and conducted a one-way repeated measure ANOVA with the mean of high-risk selections in one block as the dependent variable. The participants became cautious and began to avoid the risk-taking choices (*F*(3.42, 191.5) = 25.71, *P*<0.01) (Bonferroni; block 1>2>3 = 4 = 5) (Fig. 1A). However, close analysis of selections from deck A' and deck B' revealed a notable difference. As shown in Fig. 1B, selections from deck A' monotonously decreased as trial progressed (*F*(3.22,180.4) = 17.40, *P*<0.01) (Bonferroni; block 1>3, 4, 5; block 2>4, 5; block 3>5). On the other hand, selections from deck B' initially decreased but did not decrese towards the end of the experiment (*F*(3.24, 181.4) = 8.42, *P*<0.01) (Bonferroni; block 1>2, 3, 4, 5) (Fig. 1C). Overall, a decrease in selections from deck B' was not apparent. In addition, the standard deviations of the number of selections from deck B' became larger in the latter half of the trials. This result shows that some participants repeatedly selected cards from deck B' while others stopped selecting from this deck.

#### Card selection from deck B'

**Figure 2:**
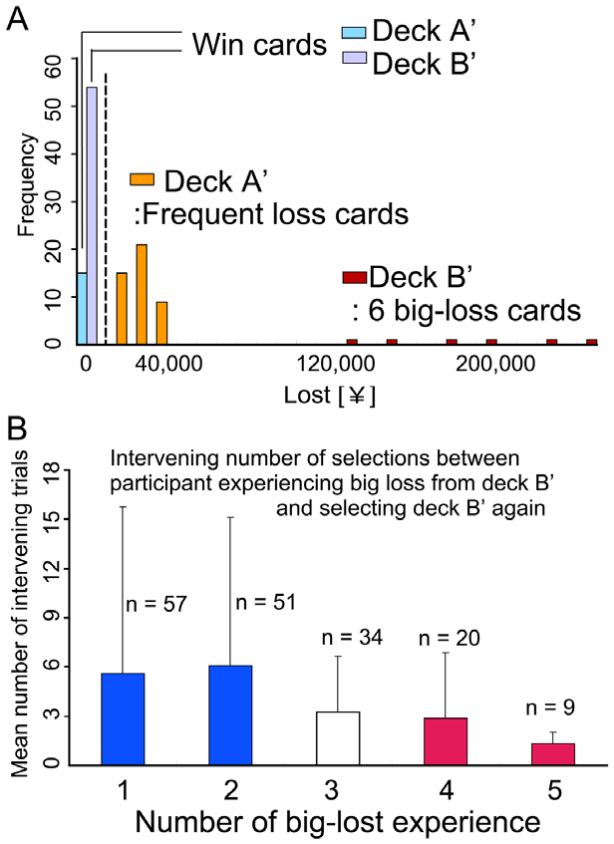
A trial-by-trial analysis based on a big loss for deck B'. (A) Comparison of losses in deck A' and deck B'. In deck A', loss cards are drawn frequently, whereas in deck B', loss cards are drawn infrequently, but include 6 big loss cards. (B) Persistence in the face of big losses from deck B': Relationship between number of big losses as a result of selecting from deck B' and number of intervening trials before selecting from the same deck B' after a big loss. Some participants tended to repeatedly select from deck B' even as they experienced big losses.

Although the total monetary gain and loss of deck B' equaled that of deck A', deck B' contained six cards that indicated extraordinarily big losses (Fig. 2A). Thus, we focused on the relationship between personality traits and behavior of making the choice of deck B'. Fig. 2B shows the results of a trial-by-trial analysis based on a big loss from deck B'. The figure plots the number of intervening trials between participant experiencing big loss from deck B' and subsequent selection from the same deck B' as a function of the number of big losses due to the deck B' selections. A big loss was defined as losing more than 100,000 Japanese yen (about 1000 US dollars). As shown in the figure, the number of intervening trials decreased as the number of big losses increased. That means participants who experienced multiple big losses tended to repeat selection from the same risky deck after a short interval.

#### Personality and “lose-persistent” behavior

**Figure 3:**
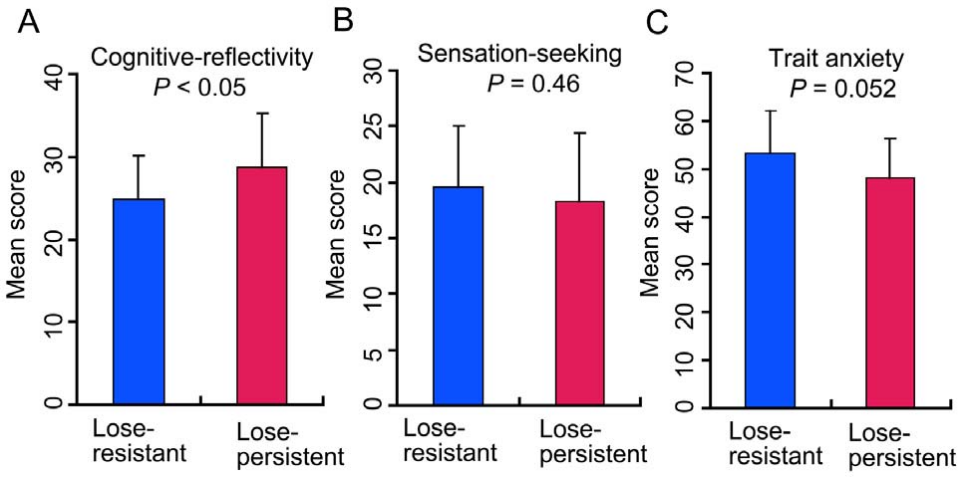
Personality traits of the lose-persistent and lose-resistant subgroups. (A) The lose-persistent subgroup had a higher cognitive reflectivity score than that of the lose-resistant subgroup. (B) There was no difference between these two subgroups in sensation seeking. (C) The lose-persistent subgroup showed a tendency of being less anxious than the lose-resistant subgroup. Statistical analysis was conducted using Student's t-test (two-tailed).

Based on the data presented in Fig. 2B, we tentatively divided participants into two subgroups: a lose-persistent group numbering 20 participants and a lose-resistant one numbering 23. The lose-persistent subgroup experienced big losses more than three times when selecting from deck B'. In contrast, the lose-resistant subgroup experienced big losses less than three times. The results are shown in Fig. 3. The participants in the lose-persistent subgroup showed significantly higher scores of cognitive reflectivity than those in the lose-resistant subgroup (*t*(41) = 2.08, *P*<0.05) (Fig. 3A). There was no systematic difference as to sensation-seeking score (*t*(41) = 0.739, *P* = 0.464) (Fig. 3B). The lose-persistent subgroup showed significantly lower scores in trait anxiety in comparison with the lose-resistant subgroup (*t*(41) = 2.00, *P* = 0.052) (Fig. 3C).

### Experiment 2

#### Risky selection

The results of experiment 1 were completely reproduced in a separate sample of participants (Fig. S1). Similar to the results of experiment 1, some participants persisted in selecting cards from deck B' after big losses. Fig. S2 shows the results of a trial-by-trial analysis of selections from deck B'. If participants experienced big losses only once or twice, they did not choose a card from this deck again after about 4.6 trials. On the other hand, participants who experienced big losses 4 or 5 times selected from the deck B' after making a few more selections from other decks (or even as their next selection).

#### Impulsiveness and risky selection: results of MFFT

**Figure 4:**
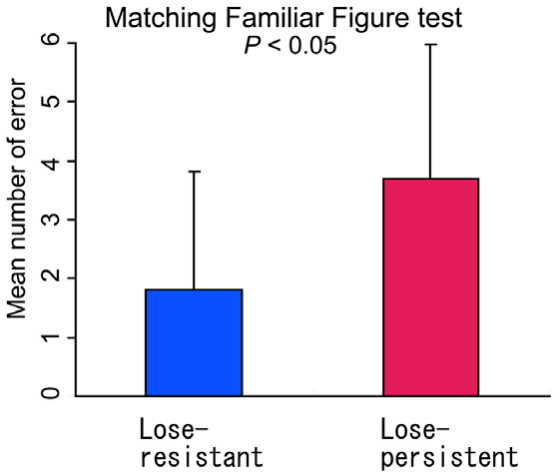
Scores of matching familiar figures test (MFFT) in the lose-resistant and lose-persistent subgroups. The mean scores and standard deviations are shown. The lose-persistent subgroup tended to score higher than the lose-resistant subgroup.

We divided participants into two subgroups as in the first experiment. The lose-resistant subgroup (n = 11) experienced big losses only once or twice, while the lose-persistent subgroup (n = 13) experienced big losses more than three times. Fig. 4 shows the mean scores of the matching familiar figures test (MFFT) together with the standard deviations in each subgroup. The lose-persistent subgroup members were more impulsive than those in the lose-resistant subgroup in this test (*t*(22) = 2.12, *P*<0.05).

#### Personality and risky selections: results of NEO-PI-R

**Figure 5:**
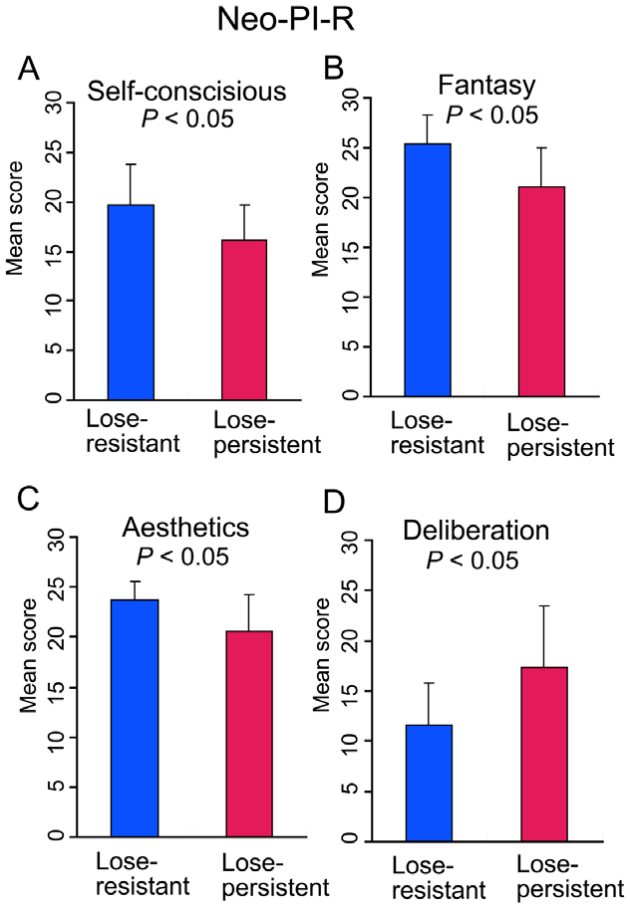
Subscales of NEO-PI-R that showed significant differences between the lose-persistent and lose-resistant subgroups. Compared with the participants in the lose-resistant subgroup, those in the lose-persistent subgroup were more deliberate, less self-conscious, and less fantasy prone (Student t-test, two-tailed).

NEO-PI-R consists of five domains. Each domain contains six subscales. Of the total of 30 subscales, four showed significant differences between participants in the lose-persistent and lose-resistant subgroups. The subscales were self-consciousness (Fig. 5A), fantasy (Fig. 5B), aesthetics (Fig. 5C), and deliberation (Fig. 5D). Participants in the lose-persistent subgroup were more deliberate (*t*(19) = 2.14, *P*<0.05), less self-consciousness (*t*(19) = 2.14, *P*<0.05), less fantasy (*t*(19) = 2.33, *P*<0.05) and less aesthetics (*t*(19) = 2.33, *P*<0.05) (Fig. 5). Other subscales did not show significant differences. Correlation analysis confirmed this finding. When we pooled data of all subjects and calculated Pearson's correlation coefficient between NEO-PI-R scores and the total number of high-risk choices (sum of selections from decks A' and B'), a significant negative correlation between fantasy subscales and high risk selections (*r* = −.414, *P*<0.01) and a significant positive correlation between deliberation subscales and high risk selections (*r* = .375, *P*<0.05) were obtained (Fig. S3). Self-consciousness did not yield significant correlations in this analysis.

## Discussion

### Experiment 1

In accordance with studies using the IGT in the traditional way of summing the choices from the two high-risk decks, most participants shifted their choices from risky ones to cautious ones [9]–[10]. Therefore, the behavior of participants in the present experiment was normal. Indeed, selections from deck A' decreased as the trials progressed. However, a big loss paradoxically worked as a positive reinforcer in about 35 percent of the participants. These participants successively selected cards from deck B' as if they wanted to experience big losses many times. These results suggest that the phenomenon of “prominent deck B” appeared even though the new versions of decks A', B', C', and D' were used.

In the personality assessments, quite unexpectedly, cognitive reflectiveness was significantly related to persistence in big losses in experiment 1. Reflectiveness is thought to be the opposite of impulsivity, and it is traditionally believed that impulsivity is related to risk-taking behaviors [5]. The reason why reflective persons tended to repeat risky choices is still unclear, but we feel the concept of reflectiveness should be re-examined. Indeed, some studies show that there is a discrepancy between reflection and rumination [30]–[31]. Reflectiveness seems to be multi-dimensional in nature.

There was no significant difference between groups as to sensation seeking behavior in experiment 1. Because sensation seeking is thought to be an important factor in addictive behaviors [32]–[33], the present finding suggests that the difference between lose-persistent and lose-resistant tendencies in might not directly relate to addictive behavioral characteristics. The trait anxiety score was lower in the lose-persistent subgroup in experiment 1. This is consistent with the previous study showing that anxiety was positively correlated with risk-avoidant decision-making [34]. However, anxiety and impulsivity are co-morbid of such mental diseases as bipolar disorder [35], eating disorder [36], and alcoholism [37]. There might be differences between clinical and non-clinical samples.

### Experiment 2

The behavioral data of the IGT was highly reproducible. In general, risky choices decreased as the trials progressed in experiment 2. However, approximately one third of the participants persisted in making risky choices from Deck B'.

Experiment 2 revealed a distinctive discrepancy between behavioral impulsiveness and self-rated deliberation. Participants who persisted in making risky choices had higher impulsiveness scores in the MFFT test but higher deliberation scores in the NEO-PI-R. Although we did not assess cognitive impulsivity using the same rating scale as used in experiment 1, the NEO-PI-R subscale deliberation acts as a substitute to the cognitive reflectiveness scale.

MFFT is widely used to detect impulsivity in relation to mental disorders such as attention deficit hyperactive disorder (ADHD) in children [38] and substance-use problems [39]. However, it is still controversial whether MFFT can detect impulsive personalities [40]–[41].

The NEO-PI-R subscales of deliberation, self-consciousness, and fantasy were related to persistence in making risky choices. Persons who persisted in making risky choices would be deliberate, less self- conscious, and less prone to fantasy. We speculate that the repetition of risky choices is related to realistic logical thinking. However, since we compared multiple items of NEO-PI-R subscales by independent statistical tests, the finding should be regarded as exploratory in nature. A more specific study on personality traits related to risky choices would help to verify the above speculation.

### General Discussion

The experiments demonstrated that a self-rating of cognitive reflectiveness or deliberation is related to persistence in making high-risk/high-return choices. Moreover, these personality tendencies were related to adherence to risk-taking choices after big losses. However, the MFFT results showed that the persistence in making risky choices was related to impulsiveness. Self-rating and MFFT might thus detect different aspects of reflectiveness/impulsivity. Self-rating is largely based on a person's conscious awareness of his or her own personality. On the other hand, MFFT might detect an unconscious level of impulsivity because study subjects were not informed that this test is used to assess impulsivity. It could be that the IGT detects an unconscious level of impulsivity. This notion is consistent with findings that the IGT reflects the function of “somatic markers” that are primarily autonomic bodily responses [9]–[10].

The next question is why unconsciously impulsive persons are consciously deliberate. One possibility is that the logical thinking leads to risky decision-making. A person may think that the loss is so large that cautious card selection can not compensate the loss. There is a report that might support this notion. In the IGT, highly educated people “paradoxically” made impulsive choices [42] and the choices made after a loss became riskier [43]. Another possibility is that experiencing a big monetary loss inspires them about the possibility of subsequent big monetary gain. For example, a previous study on video lottery terminals showed that the possibility of a near win motivated people to gamble despite the probability of monetary loss [44]. In our case, the corresponding motivating behavior would be that participants might speculate on the characteristics of the card decks and imagine a big win.

In summary, it is thought that deliberate logical thinking sometimes leads people to maintain risky decision-making behaviors and proceed to addictive behaviors such as pathological gambling in its final form. Further studies combining behavioral and electrophysiological and/or biochemical measurements would help to clarify the “paradoxical” correlation between unconscious impulsivity and conscious deliberation.

## Methods

### Participants

Normal healthy volunteers participated in the experiments (experiment 1: 57 college students: 23 males and 34 females; experiment 2: 44 college students: 21 males and 23 females). They had no history of alcohol or substance use and had been diagnosed free from any kind of mental disease. They were told about the ethical considerations before entering the study, and written informed consent was obtained from each of them. This study was conducted in accordance with the ethical code of the Japanese Psychological Society.

### Experimental Setting

The IGT developed by Bechara et al. (2000) was implemented on a personal computer [10]. We developed a Japanese version of the IGT, by converting $ to \ (Table S1). The number of trials (100) was the same as in the original version of IGT.

### Personality Assessment

In experiment 1, three kinds of personality traits were assessed: cognitive reflexivity/impulsivity [22], sensation seeking [23]–[24], and anxiety [25]–[26]. In experiment 2, the behavioral aspect of reflexivity/impulsivity was assessed by means of MFFT [29]. We used NMFFT, the more difficult version, for adults, in Japanese. NEO-PI-R was used as a measure of comprehensive personality assessment [27]–[28].

### Procedure

All experiments and personality assessments were conducted individually. Participants were invited to an experimental room and explained the aim and ethical considerations of study, one person at a time. They sat comfortably on a chair in the room and were instructed on how to operate the computer version of the IGT. Then, they were instructed to earn as much money as possible. According to the standardized IGT procedure, 100 trials were given. After completion of the IGT, personality assessments were conducted.

## Supporting Information

Figure S1 Mean number of high-risk/high-return choices across trials together with standard deviations in experiment 2. Total number of selections from deck A' and deck B' decreased across trials. The same tendency was apparent in experiment 1.
(9.15 MB TIF)

Figure S2 Relationship between number of big losses after selecting from deck B' and number of intervening trials before selecting from the same deck after big loss from deck B'. Participants tended to repeatedly select from deck B' even as they experienced big losses many times. The same tendency was apparent in experiment 1.
(2.88 MB TIF)

Figure S3 Relationship between sum of selections from deck A' and B' and score of fantasy scales (r = −.414, P<0.01)(A). Relationship between sum of selections from deck A' and B' and score of deliberation scales (r = .375, P<0.01)(B).
(0.15 MB TIF)

Table S1 Net score of deck A', B', C', and D'. These scores were by converting $ to \ in Bachara et al., 2000.
(1.13 MB TIF)
