# Supplementary figures and images for: Big Losses Lead to Irrational Decision-Making in Gambling Situations: Relationship between Deliberation and Impulsivity
Source: PLoS One. 2010 Feb 23;5(2):e9368. doi: 10.1371/journal.pone.0009368 (PMC2826400; doi:10.1371/journal.pone.0009368)

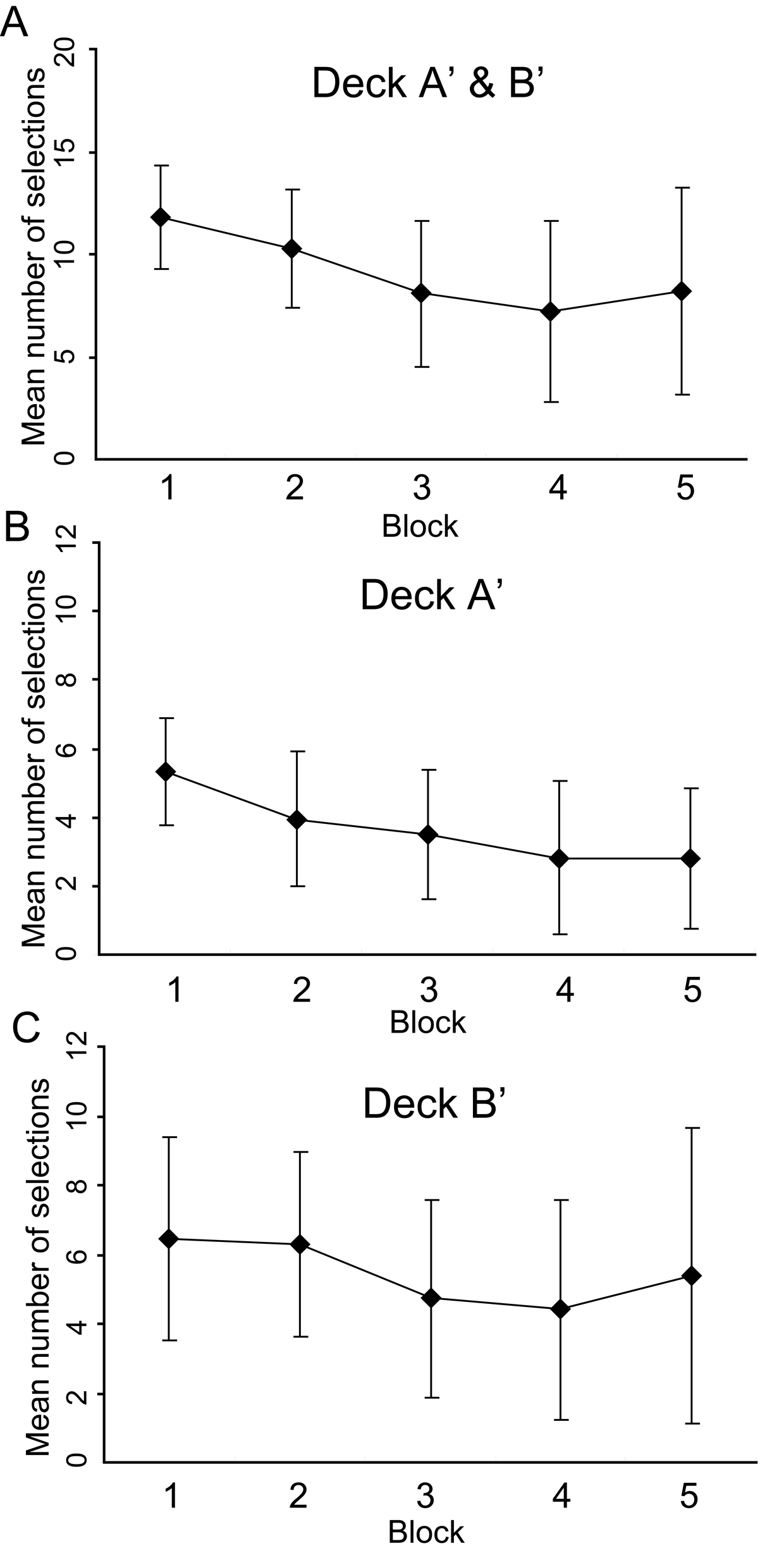

Supplement: Figure S1 — Mean number of high-risk/high-return choices across trials together with standard deviations in experiment 2. Total number of selections from deck A' and deck B' decreased across trials. The same tendency was apparent in experiment 1. (9.15 MB TIF) [file pone.0009368.s001.tif]

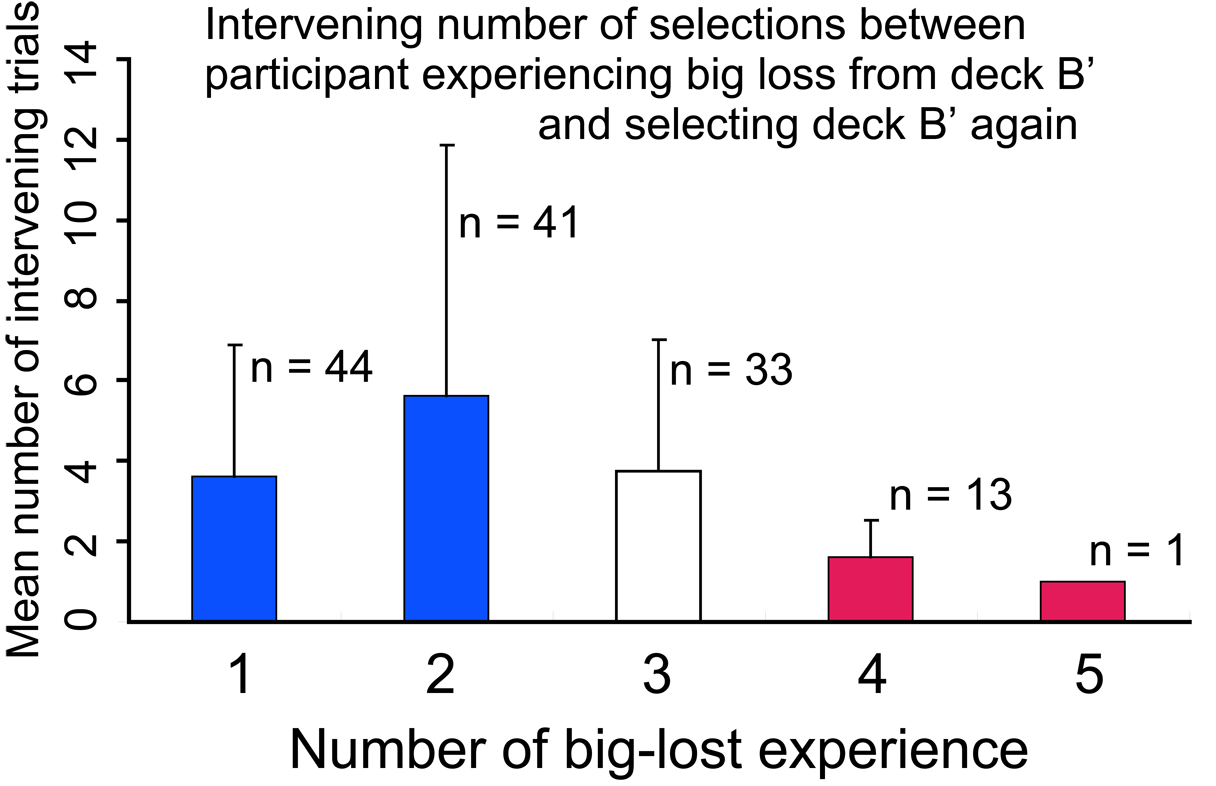

Supplement: Figure S2 — Relationship between number of big losses after selecting from deck B' and number of intervening trials before selecting from the same deck after big loss from deck B'. Participants tended to repeatedly select from deck B' even as they experienced big losses many times. The same tendency was apparent in experiment 1. (2.88 MB TIF) [file pone.0009368.s002.tif]

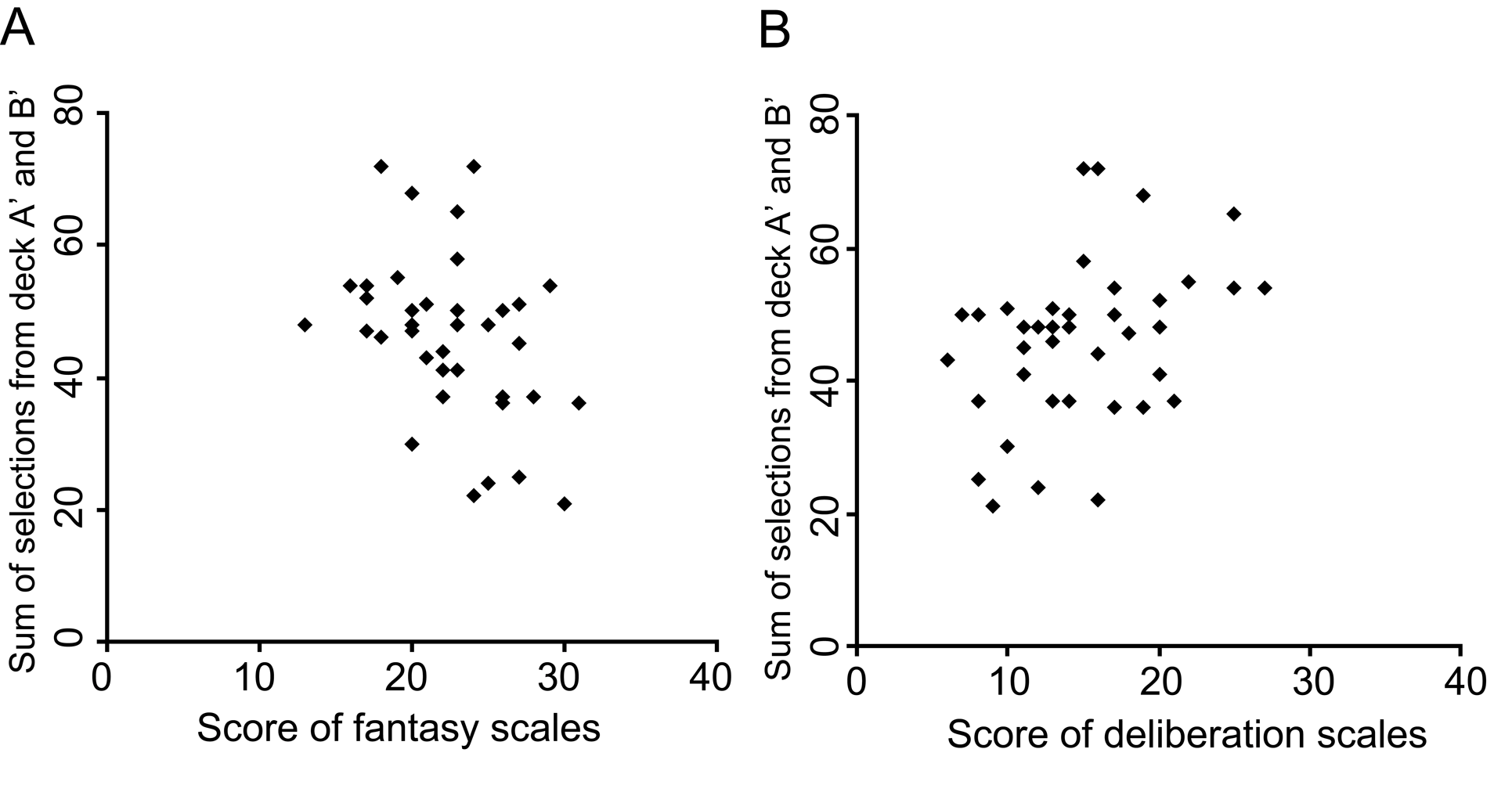

Supplement: Figure S3 — Relationship between sum of selections from deck A' and B' and score of fantasy scales (r = −.414, P<0.01)(A). Relationship between sum of selections from deck A' and B' and score of deliberation scales (r = .375, P<0.01)(B). (0.15 MB TIF) [file pone.0009368.s003.tif]

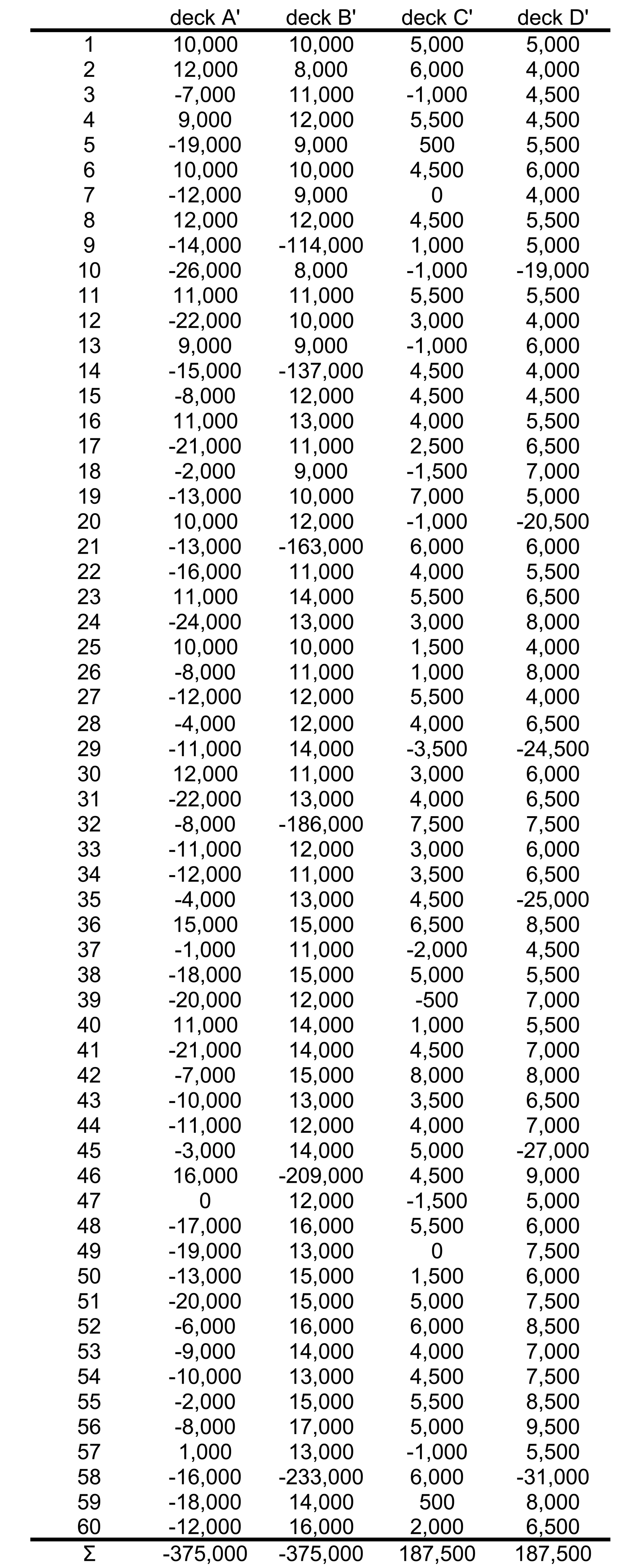

Supplement: Table S1 — Net score of deck A', B', C', and D'. These scores were by converting $ to \ in Bachara et al., 2000. (1.13 MB TIF) [file pone.0009368.s004.tif]
